# Supplementary material for: Complexome profiling of the Chlamydomonas psb28 mutant reveals TEF5 as an early PSII assembly factor
Source: Plant Cell. 2025 Mar 17;37(6):koaf055. doi: 10.1093/plcell/koaf055 (PMC12164590; doi:10.1093/plcell/koaf055)
Supplement: koaf055_Supplementary_Data [file koaf055_supplementary_data.zip › psb28_interactive.zip!/plots/intensity/qualityPlots/peptides/CAG1_Cre12.g516450.t1.2.html]

### Cre12.g516450.t1.2

Trivial name: CAG1  
SeqCoverage [median]: 24.00  
Mass: 24.29 kDa  
  
 

| Sequence | MissedCleavage | Mass [Da] | wtIntensity | muIntensity | Score |
| --- | --- | --- | --- | --- | --- |
| AGEAEYLK | 0 | 879.43 | 22531.05 | 36860.62 | 149.40 |
| ESGQALER | 0 | 888.43 | 22090.59 | 22772.40 | 142.36 |
| HATVLPMR | 0 | 923.50 | 8500.22 | 8168.28 | 142.64 |
| HATVLPMRHNVPSLDK | 1 | 1813.96 | 0.00 | 920.28 | 40.96 |
| HNVPSLDK | 0 | 908.47 | 15905.17 | 11561.94 | 149.65 |
| LQGVYSFEEK | 0 | 1198.59 | 14942.93 | 8187.15 | 130.75 |
| YTELAGEHK | 0 | 1046.50 | 7868.71 | 4466.88 | 114.50 |
| YTELAGEHKGIMK | 1 | 1475.74 | 426.59 | 7801.53 | 85.55 |
|  |  |  | 92265.3 | 100739.1 |  |
